# Melanotic neuroectodermal tumor of infancy of the shoulder in a 4-month-old infant: Case report

**DOI:** 10.1016/j.radcr.2026.04.079

**Published:** 2026-06-02

**Authors:** Ayoub Boutahar, Karima Atarraf, Sanae Bouramdan, Mohamed Tazi Charki, Hicham Abdellaoui, My Abderrahman Afifi

**Affiliations:** a Faculty of Medicine and Pharmacy, University Abdelmalek Essaadi, Tangier, Morocco; b Department of Pediatric Surgery, The University Hospital Center Mohammed VI, Tangier, Morocco; c Faculty of Medicine and Pharmacy, University Sidi Mohamed Ben Abdellah, Fez, Morocco; d Department of Pediatric Traumatology and Orthopedics, The University Hospital Center Hassan II, Fez, Morocco; e Department of Pediatric Oncology, The University Hospital Center Hassan II, Fez, Morocco

**Keywords:** Melanotic neuroectodermal tumour of infancy, Melanotic progonoma, Shoulder, Immunohistochemistry, Neoadjuvant chemotherapy

## Abstract

Melanotic neuroectodermal tumor of infancy (MNTI) is a rare, benign yet locally aggressive tumor, that typically arises in the maxillofacial region in young infants. We report a 4-months-old male child presented with 2 months history of a swelling in the right shoulder. Imaging revelated a mass in close contact with the aponeurosis of the deltoid and biceps muscles, initially suggestive of a sarcomatous origin. Immunohistochemistry study was done confirming the diagnosis of MNTI. Neoadjuvant chemotherapy reduced tumor volume enabling safe and complete resection preserving neurovascular and shoulder function. MNTI should be considered in the differential diagnosis of aggressive soft tissue or bone masses in infants, even in atypical locations such as the shoulder.

## Introduction

Melanotic neuroectodermal tumor of infancy (MNTI) is a rare, benign yet locally aggressive tumor typically affecting infants under 1 year of age. A small number of cases have been reported in older children and in adults. No predilection in gender was described. Known by various synonyms (congenital melanocarcinoma, retinal anlage tumor, pigmented congenital epulis or melanotic progonoma), and it is generally accepted to be neural crest in origin [[1], [2], [3], [4], [5], [6]].

While 90%-95% of cases arise in the maxillofacial region (mainly maxilla), extra-maxillofacial localizations -including skull, mandible, cranial vault, mediastinum, long bones of the extremities, and rarely genital organs- have been reported [7,8]. Shoulder involvement remains exceptional.

The treatment of choice for this tumor is surgical excision. Because of its relatively high recurrence rate, cases of MNTI should be monitored closely during the postresection period and beyond. Metastasic potential is low (approximately 5%), and the role of adjuvant chemotherapy or radiotherapy remains unproven [9,10].

We report a 4-month-old male infant with proximal humerus melanotic progonoma, initially suspected to be sarcomatous, treated with neoadjuvant chemotherapy followed by surgical resection.

## Case presentation

A 4-months-old male child presented with a progressive right arm swelling noticed by his parents at 2 months of age. There was no fever, and the child was fully immunized with normal developmental milestones. General and systemic examinations was unremarkable. Local examination revealed a well-defined, firm, non-pulsatile mass measuring 4*3 cm along the long axis (Fig. 1).

**Fig. 1:**
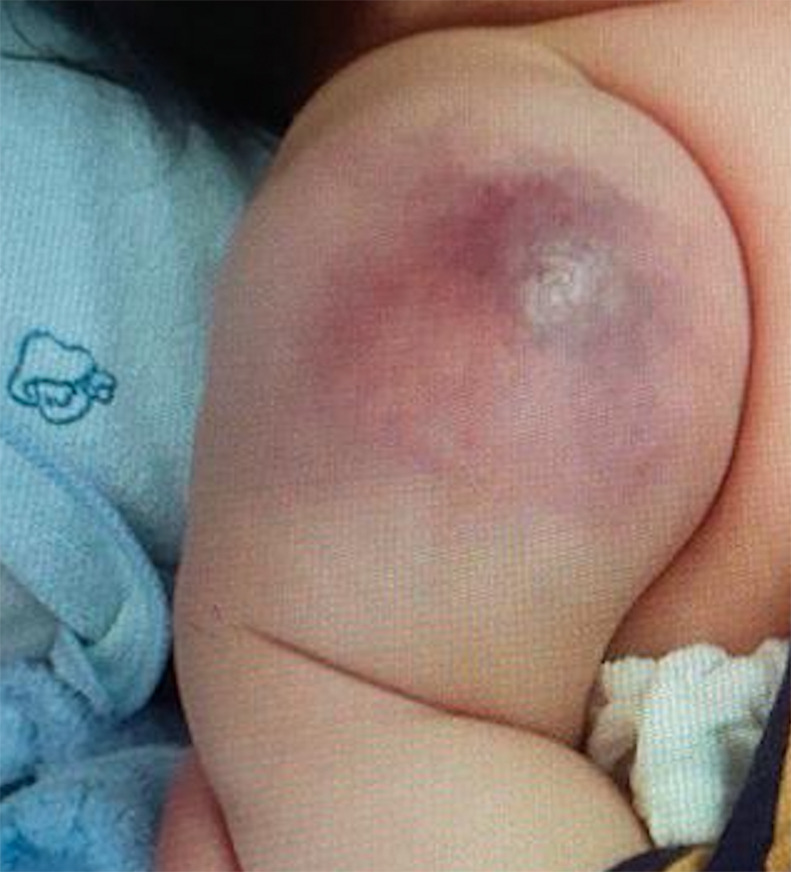
Clinical appearance of a 4 × 3 cm mass along its long axis.

The X-ray showed no bony abnormalities. Ultrasound demonstrated well circumscribed heterogenous soft tissue mass measuring 3,5*2,5 cm without Doppler hypervascularity (Fig. 2). MRI confirmed an oval extra-aponeurotic mass subcutaneous mass measures 5 cm * 3,5 cm with irregular contours, intense contrast enhancement, central necrotic, and close contact with deltoid and biceps aponeurosis - suggesting possible invasion - but separated from the humeral bone by the muscular and neurovascular structures (Fig. 3). So, a sarcomatous origin was suspected. All imaging examinations were performed within a short time frame, minimizing the likelihood of true lesion size variation, and this minor variations may be explained by differences in imaging techniques and acquisition planes.

**Fig. 2:**
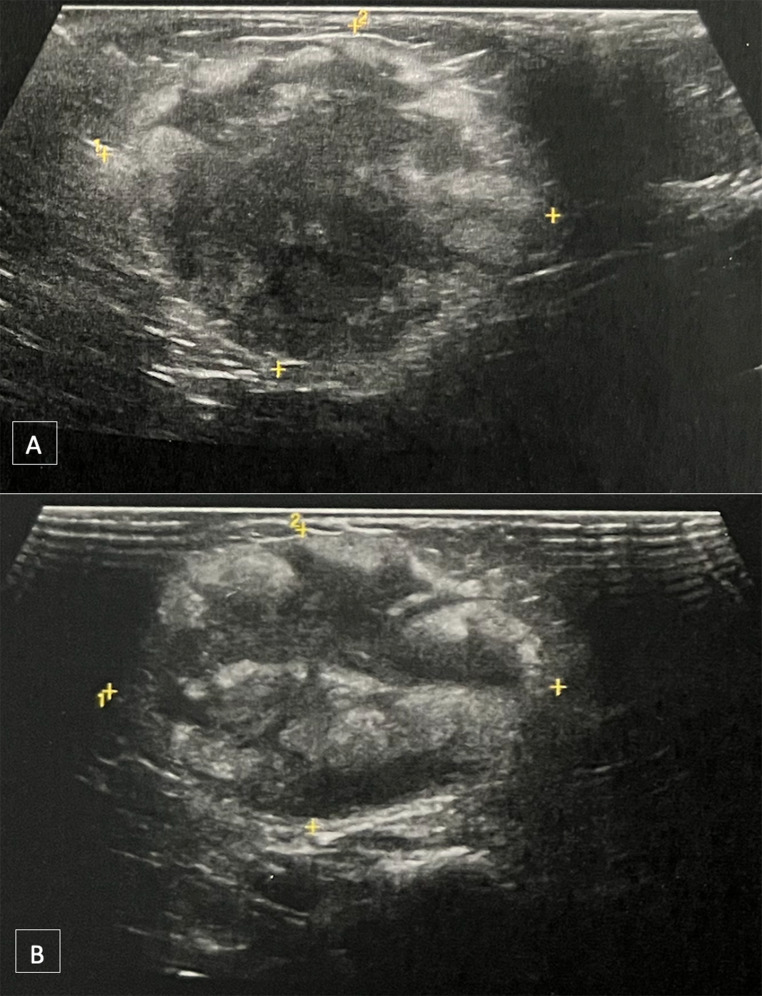
Ultrasound: well circumscribed heterogenous soft tissue mass.

**Fig. 3:**
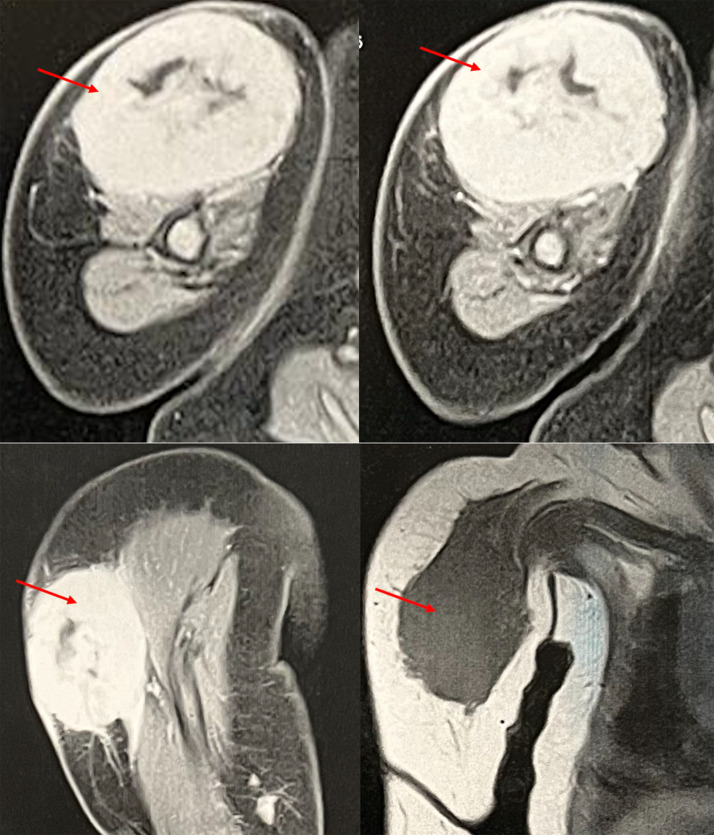
MRI: Oval extra-aponeurotic mass subcutaneous mass measures with irregular contours, intense contrast enhancement, central necrotic.

A biopsy of the tumor revealed small round tumor cells with hyperchromatic nuclei, scant basophilic cytoplasm, focal melanin pigment within thickened trabeculae of a fibrous stroma. Immunohistochemistry showed positivity for CK, synaptophysin, chromogranin, CD99 and THMB45; Negative for of PHOX2B (excluding neuroblastic tumor), melan A, CD45, desmin, myogenin, PLAP or S100. FISH ruled out Ewing sarcoma. The diagnosis was melanotic neuro ectodermal tumor of infancy (MNTI).

The infant received neoadjuvant chemotherapy per SMOP-NBL1 protocol (2 cycles: CADO/CARBO - VP16), reducing tumor volume by 45% on follow-up local examination and MRI (Fig. 4). Surgical exploration revealed a mass adherent to brachialisand biceps brachii muscles, pectoralis major tendon, triceps muscle and musculocutaneous nerve, but sparing the radial nerve (Fig. 5). Complete resection included portions of involved muscles and adjacent periosteum, followed by pectoralis major reinsertion, tension-relieving incisions, and layered closure. 6-month follow-up showed complete wound healing, full range of motion and without recurrence.

**Fig. 4:**
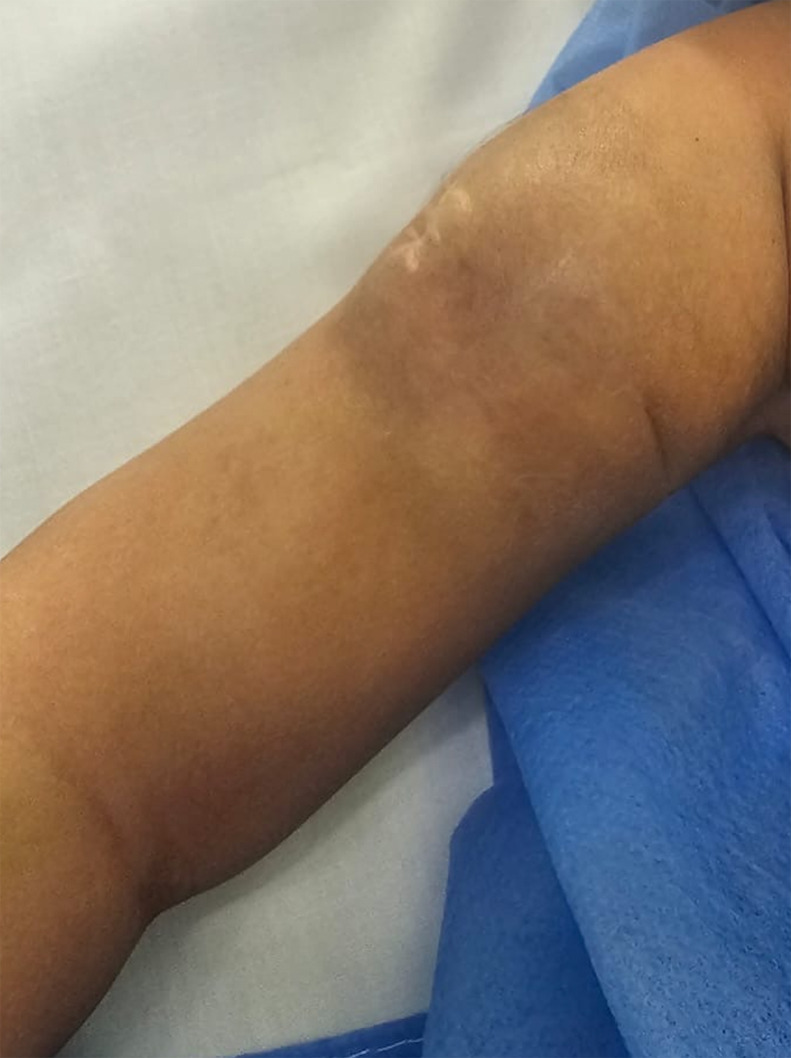
Clinical appearance of a 3 × 2 cm mass after neoadjuvant chemotherapy.

**Fig. 5:**
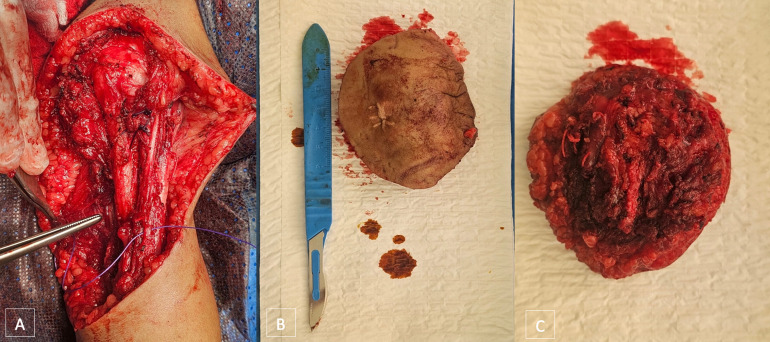
(A) Intraoperative view after complete resection of the tumor. (B, C) Complete resection included portions of involved muscles and adjacent periosteum.

## Discussion

First described by Krompecher in 1918 as “congenital melanocarcinoma”, MNTI has been known by multiple synonyms (retinal anlage tumor, melanotic progonoma). Borello and Gorlin proposed the current name (melanotic neuroectodermal tumor of infancy) in 1966, linking it to neural crest origin via elevated urinary VMA levels [6]. Affecting mainly infants <1 year and has male predominance, 90%-95% of MNTI occur in the craniofacial region particularly the maxilla [11,12]. Extra-maxillary sites (skull, mandible, long bones, epididymis, mediastinum, extremities) are rare (5%-10%).

Shoulder localization, as in this case, is exceptional and underscores the need for broad differential diagnosis of rapidly growing pediatric soft tissue masses (neuroblastoma, Ewing sarcoma, Burkitt lymphoma). Clinically painless, non-ulcerated, and pigmented (blue or pink appearance), MNTI appears expansive [13]. Conventional radiographs and CT images are useful (but not specific) for assessment of the lesion extension. The usual image is a large cavity of osteolysis with well-delineated form without any specificity. The image could be less marked, from a simple thinning of the cortical to real bone destruction, with or without osteogenic reaction. MRI with gadolinium gives the clearest images, allowing the distinction of the tissue components of this tumor, it reveals a hypodense mass, within a heterogeneous well-delineated lesion on T1- and T2-weighted images. The empty circulatory areas observed in hemangiomas are not present in the MNTI [14].

Cytological analysis requires differentiating MNTI from other small round cell tumors in infants (neuroblastoma, Ewing sarcoma, and Burkitts lymphoma), especially when small cells predominate. Melanin pigmentation and/or pigmented cells render the cytology characteristic [15].

Histologically [16], MNTI displays a biphasic pattern: two cell types with distinct morphology and immunohistochemical profile. Tumor cells form irregular alveolar, tubuloglandular and fissured patterns amid dense fibroblastic stroma. The large melanin-producing polygonal epithelioid cells had vesicular nuclei, prominent nucleoli and eosinophilic cytoplasm, while the small round neuroblast-like cells were surrounded by the epithelioid cells and showed hyperchromatic nuclei and scanty cytoplasm [17].

Neoadjuvant chemotherapy reduced tumor volume be 45% here, facilitating wide resection despite muscle and nerve adherence, and preserving radial nerve function. Surgery remains curative (recurrence 10%-20%), with low metastasis risk. Cases with MNTI that are not amenable to surgical management alone may receive other modes of treatment [18]. In general, this may be chemotherapy alone, chemotherapy with radiotherapy, chemotherapy before and after the surgical treatment, radiotherapy and surgical treatment or a combination of all. Chemotherapy may serve as an alternative or adjuvant option in the treatment of widely extended MNTIs [12,19]. Long-term follow-up is mandatory for functional shoulder outcome.

## Conclusion

MNTI, though histologically benign, exhibits rapid growth and local invasiveness requiring early diagnosis. Neoadjuvant chemotherapy facilitated safe resection in this rare shoulder localization, preserving neurovascular and shoulder function. Long-term clinical and radiological follox-up is essential to monitor recurrence and growth-related complications.

## Data availability statement

The data supporting the findings of this study are available upon request from the corresponding author.

## Author contribution statement

All authors have read and approved the final version of the manuscript.

## Patient consent

Written informed consent for publication of this case report and accompanying images was obtained from the patient’s parents. A copy of the written consent is available for review by the Editor-in-Chief of this journal upon request.
